# Comments on the article “Effectiveness and safety of tofacitinib in rheumatoid arthritis: a cohort study”

**DOI:** 10.1186/s13075-018-1651-7

**Published:** 2018-08-29

**Authors:** Thuy Nhu Thai, Ghadeer K. Dawwas

**Affiliations:** Department of Pharmaceutical Outcomes and Policy, College of Pharmacy, University of Florida, Gainesville, Florida USA

We read with interest the study published by de Ávila Machado et al. regarding the comparative effectiveness and safety of tofacitinib and non-tumor necrosis factor (TNF) inhibitors among patients with rheumatoid arthritis (RA) [1]. Although the study findings are very interesting, there are several concerns in regard to the study design. First, the study used an exposure definition that caused RA disease severity to be heterogeneous between and within the comparison groups. According to RA treatment guidelines, patients at an early stage of RA are recommended to start a combination of DMARDs, or to initiate TNF inhibitors, or non-TNF inhibitors after the failure of DMARDs monotherapy. On the other hand, patients with an established RA are recommended to use a combination of DMARDs, DMARDs with TNF inhibitors, DMARDs with non-TNF inhibitors, or DMARDs with tofacitinib [2]. Therefore, the exposure definition used in this study may reflect RA patients at different stages of disease severity which has the potential of introducing bias in the observed estimates.

For example, to initiate tofacitinib, patients have to fail the other treatment approaches causing them to be at an advanced RA stage when compared with those in the DMARDs group who are more likely to have used methotrexate only before. Additionally, it was not clear why the authors grouped together patients who combined TNF inhibitors with DMARDs along with patients who used TNF inhibitors alone. This exposure group might be comprised of two different patients' populations since it is often recommended that patients combine TNF inhibitors and DMARDs after treatment failure with TNF inhibitors alone [2].

Second, the medication possession ratio (MPR) is known to be a valid measure of adherence of a single medication. However, MPR tends to overestimate adherence when patients use more than one medication [3]. Accordingly, the observed adherence of patients who used two or more DMARDs are more likely to be overestimated. The proportion of days covered is suggested as a better alternative measure of adherence in this situation [3]. Third, the models evaluating the effectiveness and safety omitted the adjustment of important variables. For example, the authors did not adjust for current methotrexate use which may impact the observed treatment effectiveness or factors that may increase the risk of infections such as human immunodeficiency virus(HIV) or the use of immunosuppressant drugs.
