# Supplementary material for: Comments on the article “Effectiveness and safety of tofacitinib in rheumatoid arthritis: a cohort study”
Source: Arthritis Res Ther. 2018 Aug 29;20:198. doi: 10.1186/s13075-018-1651-7 (PMC6116480; doi:10.1186/s13075-018-1651-7)
Supplement: Related texts

# Reply: Authors’ response

**Authors:** Marina Amaral de Ávila Machado, Cristiano Soares de Moura, Steve Ferreira Guerra, Michal Abrahamowicz, Sasha Bernatsky

We thank Thai and Dawwas for their interest in our work on the effectiveness and safety of tofacitinib in RA [1] and we are pleased to clarify some aspects of our study.

The first comment concerned comparability of exposure groups. We selected patients previously treated with methotrexate and other newly dispensed DMARDs, biologics, and tofacitinib between 2011 and 2014. We applied a new-user design, such that all patients in our study were initiating a second RA therapy after failure or intolerance of methotrexate. We defined four comparison groups: DMARDs, TNF inhibitors with or without DMARDs, non-TNF biologics with or without DMARDs, and tofacitinib with or without DMARDs. Non-TNF biologics comprised the reference group, given our particular interest in assessing subsequent therapies in RA. We allowed the groups to be concomitantly exposed to DMARDs since, in the real world, it is not uncommon for these drugs to be given in combination, for example with anti-malarial drugs or other DMARDs. Previous studies have applied similar approach [4, 5]. Upon further review of our paper, we noticed an inconsistency in the fourth sentence of the second paragraph of the Methods section that may have contributed to a misunderstanding. We stated that “We selected individuals with no use of these medications any time before cohort entry (minimum 12 months), although previous use of DMARDs was allowed for individuals in the biologic and tofacitinib groups.” In fact, patients in the biologic and tofacitinib groups who had been prescribed DMARDs before cohort entry were considered in the DMARD group.

Regarding the second point about the adherence of patients on combination therapy with DMARDs, we clarify that the medication possession ratio (MPR) was measured separately for the index DMARD (identified at the pharmacy claim at cohort entry) and for methotrexate. Then, a given patient was considered highly adherent for the combined therapy if the MPR was ≥ 80% for the DMARD or for methotrexate.

Finally, the authors questioned the omission of some potential confounders in the models. We did not adjust the effectiveness model for current methotrexate use because the addition of methotrexate between 4 and 12 months of follow-up was part of the outcome definition (criterion 3 of the algorithm). Regarding infection risk, the safety models were adjusted for previous and current use of glucocorticoids, and previous hospitalized infections [6]. We also adjusted for Charlson comorbidity index, which includes AIDS/HIV.
